# The interleukin-1 receptor type-1 in disturbed flow-induced endothelial mesenchymal activation

**DOI:** 10.3389/fcvm.2023.1190460

**Published:** 2023-07-19

**Authors:** Evan Kidder, Meleah Pea, Siyuan Cheng, Satya-Priya Koppada, Suren Visvanathan, Quartina Henderson, Moe Thuzar, Xiuping Yu, Mabruka Alfaidi

**Affiliations:** ^1^ Department of Internal Medicine-Division of Cardiology, Louisiana State University Health Sciences Center at Shreveport, Shreveport, LA, United States; ^2^ Department of Urology, Louisiana State University Health Sciences Center at Shreveport, Shreveport, LA, United States; ^3^ Department of Biochemistry and Molecular Biology, Louisiana State University Health Sciences Center at Shreveport, Shreveport, LA, United States; ^4^ Department of Pathology and Pathobiology, Louisiana State University Health Sciences Center at Shreveport, Shreveport, LA, United States; ^5^ Feist-Weiller Cancer Center, Louisiana State University Health Sciences Center at Shreveport, Shreveport, LA, United States; ^6^ Center for Cardiovascular Diseases and Science (CCDS), Louisiana State University Health Sciences Center at Shreveport, Shreveport, LA, United States

**Keywords:** disturbed flow, endothelial-to-mesenchymal, activation, atherogenic, interleukin-1 receptor type-1

## Abstract

**Introduction:**

Atherosclerosis is a progressive disease that develops in areas of disturbed flow (d-flow). Progressive atherosclerosis is characterized by bulky plaques rich in mesenchymal cells and high-grade inflammation that can rupture leading to sudden cardiac death or acute myocardial infarction. In response to d-flow, endothelial cells acquire a mesenchymal phenotype through endothelial-to-mesenchymal transition (EndMT). However, the signaling intermediaries that link d-flow to EndMT are incompletely understood.

**Methods and Results:**

In this study we found that in human atherosclerosis, cells expressing SNAI1 (Snail 1, EndMT transcription factor) were highly expressed within the endothelial cell (EC) layer and in the pre-necrotic areas in unstable lesions, whereas stable lesions did not show any SNAI1 positive cells, suggesting a role for EndMT in lesion instability. The interleukin-1 (IL-1), which signals through the type-I IL-1 receptor (IL-1R1), has been implicated in plaque instability and linked to EndMT formation *in vitro*. Interestingly, we observed an association between SNAI1 and IL-1R1 within ECs in the unstable lesions. To establish the causal relationship between EndMT and IL-1R1 expression, we next examined IL-1R1 levels in our *Cre-lox* endothelial-specific lineage tracing mice. IL-1R1 and Snail1 were highly expressed in ECs under atheroprone compared to athero-protective areas, and oscillatory shear stress (OSS) increased IL-1R1 protein and mRNA levels *in vitro*. Exposure of ECs to OSS resulted in loss of their EC markers and higher induction of EndMT markers. By contrast, genetic silencing of IL-1R1 significantly reduced the expression of EndMT markers and Snail1 nuclear translocation, suggesting a direct role for IL-1R1 in d-flow-induced EndMT. *In vivo*, re-analysis of scRNA-seq datasets in carotid artery exposed to d-flow confirmed the IL-1R1 upregulation among EndMT population, and in our partial carotid ligation model of d-flow, endothelial cell specific IL-1R1 KO significantly reduced SNAI1 expression.

**Discussion:**

Global inhibition of IL-1 signaling in atherosclerosis as a therapeutic target has recently been tested in the completed CANTOS trial, with promising results. However, the data on IL-1R1 signaling in different vascular cell-types are inconsistent. Herein, we show endothelial IL-1R1 as a novel mechanosensitive receptor that couples d-flow to IL-1 signaling in EndMT.

## GRAPHICAL ABSTRACT

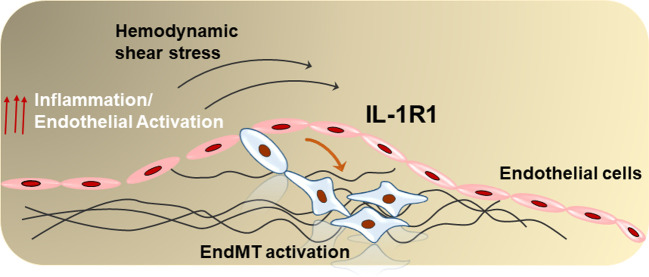

## 1. Introduction

Progressive unstable atherosclerosis is characterized by a thin fibrous cap, low smooth muscle and high mesenchymal cell content, with a high grade of inflammation, and is the leading cause of cardiovascular disease-related death (1). Despite decades of research on atherosclerosis, the molecular mechanism(s) of progressive unstable atheroma are only partially understood. Human studies have suggested that the cellular composition of plaques is a predictor of plaque instability (2). A sub-endothelial cell population in unstable lesions with mesenchymal features is usually associated with the incidence of atheroma in human aortas (3). Mouse studies using endothelial-specific lineage tracing and single cell RNA sequencing (scRNA-seq) systems demonstrate that endothelial cells (ECs) can undergo mesenchymal transition (EndMT) and form a substantial number of these sub-endothelial cells (4–7). EndMT, which develops in areas of disturbed blood flow (d-flow) (6), has been implicated in the progression of atherosclerosis (8) where EndMT plays an important role in plaque destabilization by promoting local inflammation (8, 9). Despite this, the molecular mechanism by which EndMT develops under d-flow areas remains incompletely understood.

The interleukin-1 (IL-1) signaling pathway has been shown to play a role in the severity of atherosclerosis progression and EndMT formation (10). IL-1 is therefore a suitable therapeutic target for modulation of EndMT that enhances atherosclerosis stability. The major clinically concluded CANTOS study tested the therapeutic effect of the anti-IL-1β antibody, Canakinumab, in a group of patients after myocardial infarction, and showed 31% reduction in cardiovascular and all-cause mortality in certain, but not all, groups of patients (11). While global inhibition of IL-1 signaling in this study showed some promising results, a number of patients developed off-target immunosuppression effects (12), limiting the global targeting of IL-1 in atherosclerosis. Another limitation in the field is that despite extensive studies, there is no clear principal cell type responsible for IL-1 signaling in human progressive atherosclerosis.

The term Interleukin-1 (IL-1) refers to 2 cytokines, IL-1α and IL-1β, which signal exclusively through a common receptor, IL-1 receptor type I (IL-1RI) (13). IL-1 is implicated in several aspects of plaque instability (14, 15) and IL-1 signaling is linked to EndMT formation *in vitro* (16). Global inhibition of IL-1 in atherosclerosis has been extensively investigated (12, 17). However, the data on IL-1R1 is inconsistent. In mice, either global or myeloid specific deletion of IL-1R1 reduces atherosclerosis (18), whereas cell specific deletion of IL-1R1 in vascular smooth muscle cells increases atherosclerosis and make lesions more unstable (19–21). However, in humans with coronary atherosclerotic disease it is still unclear where and at which stage of the disease IL-1R1 is expressed.

Previously we and others have shown that in human coronary atherosclerotic plaques IL-1 is predominantly expressed within the endothelium (22–24), suggesting a role for endothelial IL-1 signaling in the pathogenesis of atherosclerosis. Our published data demonstrated enhanced signaling activation of interleukin-1 receptor predominantly within the endothelium of progressive human atherosclerotic plaques (22, 24). In addition, we showed that this activation is induced by d-flow *in vitro* and *in vivo* (25). Despite all of that, the signaling roles of IL-1R1 in the endothelium have yet to be extensively investigated.

Taking into account the important role of EndMT in progressive human atherosclerosis and IL-1R1 signaling roles, in this study, we directly assessed IL-1R1 expression and linked it to EndMT in human atherosclerotic plaques. Furthermore, we utilized our endothelial specific IL-1R1 KO mice to determine the endothelial roles of IL-1R1 in d-flow-induced EndMT. Our study should aid in developing future strategies for targeting the IL-1R1 within the endothelium to limit atherosclerosis progression and maintain lesion stability.

## 2. Materials and methods

The authors declare that all the data supporting the current study is available either within the article or online data supplements. All reagents were provided by Fisher Scientific, USA, unless otherwise stated.

### 2.1. Cell culture, siRNA transfection, and isolation of Il-1r1 knockout cells

HAOECs (Human aortic endothelial cells) (Lot # 1819; Cat. #: 304-05A; Sigma-Millipore) were cultured as previously described (25) and used between passage 6–8. The cells were grown in 10% v/v MCDB131 media (GenDEPOT; CM034-300) supplemented with fetal bovine serum (Sigma; Cat. # F0926), 100 IU Penicillin-Streptomycin (Sigma, Cat # P4333), 2 mM Glutamax (Gibco, Cat. #35050), 0.06 mg/ml Heparin Sodium Salt (Thermo-Scientific, Cat #41121-0010), and 100 µg/ml Corning Endothelial Cell Growth Supplement (Cat. # CB-40006B).

IL-1R1 was knocked down using SMARTPool siRNA (IL-1R1 siRNA, 50 nM, Cat. #L-005188, Dharmacon™) and Lipofectamine 3000 transfection reagent (Invitrogen, Cat. # L3000-015) according to the manufacturer's instructions. Transfection was conducted in 1× OPTIMUM Reduced Media (Gibco; # 31985-070) for 3 h.

Mouse aortic endothelial cells from IL-1R1^fl/fl^ were isolated as previously described (25). Purity of endothelial cell isolation was assessed with double immunostaining for CD31 (Anti-CD31, abcam, ab9498) and smooth muscle actin (Anti-Actin SMA-FITC antibody CLONE 1a4, Sigma, F3777). Cell transformation was conducted in LSU Molecular-sub-core. The cells were divided into two 10 cm^2^ plates and at 70% confluence the cells were treated with either Ad-CMV-GFP (VECTOR BIOLABs, cat. # 1060, 1 × 10^8^ PFU/ml) as wild-type (WT) controls, or Ad-GFP-2A-iCre (VECTOR BIOLABS, cat. # 17772, 2 × 10^8^ PFU/ml) as IL-1R1 knockout (IL-1R1 KO) cells. The transduction was induced for 72 h. and the GFP positive cells were sorted using flow cytometry at the LSU Research Core Facility. The efficiency of IL-1R1 deletion was assessed by measuring the IL-1R1 mRNA expression using qRT-PCR.

### 2.2. Oscillatory shear stress experiments

Oscillatory shear stress (OSS, 1 dynes/cm^2^ with 1 s switching time; flow rate 2.38 ml/min) was induced by the Ibidi parallel plate flow system as previously described (26). The cells at a seeding density of 1 × 10^6^ were plated in channel slides (µ-slide I 0.6 luer; 150 µl per slide) to allow to adhere for 2 h. in 1% (v/v) MCDB131 media. Confluent slides were placed under the flow, Perfusion Set RED, using Ibidi pump system (IBIDI USA, INC) for 24 h.

### 2.3. Partial carotid ligation experiments

All the animal breeding and experiments were approved by the institutional animal care and use committee (Louisiana State University Health Sciences Center at Shreveport, protocol # P-23-003 to MA). ApoE (apolipoprotein E-null) knockout (ApoE^−/−^), IL-1R1^fl/fl^ (stock No. 028398), ROSA-Stop-Flox-YFP (stock No. 006148), and VE-cadherin-CreERT^tg^ (stock No. 006137) mice were all purchased from the Jackson Laboratory. IL-1R1^fl/fl^ were cross-bred in our laboratory with VE-cadherin-CreERT^tg^ and ApoE^−/−^ mice to create IL-1R1^iEC−KO^ (VE-cadherin-CreERT, IL-1R1^fl/fl^, ApoE^−/−^) mice. The inducible iEC lineage-tracing mice were generated in house by backcrossing (VE-cadherin-CreERT2^tg/+^, ROSA-Stop-Flox-YFP^tg/+,^ ApoE^−/−^) and used for immunofluorescence.

The male sex is at higher risk for atherosclerotic heart disease (27). Therefore, only male mice were used in the current study. Male mice at 8–10-weeks of age of IL-1R1^iEC−KO^ and their littermate controls were injected intraperitoneally with tamoxifen (Sigma, cat. # T-5648) in peanut oil (0.1 ml at concentration of 45.5 mg/kg ∼1 mg/mouse) daily for 5 consecutive days. The mice (number in the figure legends) were allowed to recover for 2 weeks before the surgery was conducted. To induce d-flow, partial carotid ligation was performed as previously shown (25, 28). Briefly, the anesthesia was induced, and the frontal area of the mouse neck was cleaned and opened by a midline incision. The left carotid artery was dissected by blunt dissection and the three branches (internal carotid, external carotid, and occipital branch) were ligated with 0–6 silk sutures, leaving the superior thyroid branch intact. The skin incisions were sutured with 0–5 sutures and the mice were hydrated with 0.5% normal saline (1 ml) and analgesics were administered subcutaneously using Carprofen (5 mg/kg; Ceva, USA). 2-week post-carotid ligation, mice were euthanized and left, and right carotid arteries were collected for RNA analysis as shown (28). The inducible endothelial deletion of IL-1R1 were verified in mRNAs isolated from carotid intima using TRizol flush method and that is after tamoxifen injection, and we measured the intimal expressions of IL-1R1, CD31, SNAI1 mRNA as we previously described (25, 28).

### 2.4. Western blotting

Gel electrophoresis was performed on denatured protein samples using the Invitrogen™ Mini Gel Tank and NuPAGE™ 4%–12%, Bis-Tris, 1.0–1.5 mm, Mini Protein Gels, as previously described (29). Samples of 25 µl protein lysates were loaded per well and allowed to run in MOPS SDS 1× running buffer for 45 min at 200 V. Wet transfer was performed in 1× Invitrogen™ Blot™ Transfer Buffer for 1 h. at 20 V. Once the proteins were transferred to the PVDF membranes (Bio-Rad, Cat. #1620177), the membranes were blocked in 5% (w/v) non-fat dry milk in 0.01% (v/v) TBST (Tween-Tris-buffered saline) for 1 h. The membranes were incubated with the desired primary antibodies (Supplementary Table SI), diluted as appropriate in 1% (w/v) BSA/PBS (Bovine serum albumin, Sigma, Cat. # A2153) overnight at 4°C with gentle agitation. The membranes were then incubated with the secondary antibodies (Peroxidase Affinity Pure Goat Anti-mouse or Anti-Rabbit IgG, Jackson ImmunoResearch, Cat. # 115-035-003) for 1 h.

Detection was performed by chemiluminescence using SuperSignal™ Chemiluminescence substrate (Cat. # 34580) and scanned using the Bio-Rad ChemiDoc™ MP Imaging System. For quantification, protein densitometry data were quantified using image j software. Fold changes in the protein expressions were measured relative to the loading control and to the static condition as previously demonstrated (25, 28).

### 2.5. Immunohistochemistry

Human coronary artery samples were collected in the pathology department at LSU Health—Shreveport. The collection was approved by the Local LSU Health Research Ethics Committee. The samples were stored in 10% v/v formalin until processing. The tissue samples were Paraffin-embedded and sectioned at 5 µm on slides. Sections of human coronary atherosclerotic plaques were blindly stained and scored for lesion stability criteria by a pathologist at LSU Health—Shreveport.

The stable, moderately stable, and unstable lesions were stained for H&E staining, which was performed using H&E Stain Kit (abcam, ab245880) according to the manufacturer's instructions. For immunofluorescence staining, it was performed as previously described (25). After rehydration, sections were heated in Antigen Unmasking Solution, Citric Acid Based (Cat. # H-3300) for 10 min in a pressure cooker to retrieve antigen epitopes, then allowed to cool down for 30 min at room temperature before they were blocked in 10% (v/v) horse serum in 1% w/v BSA, and stained with the following primary antibodies at 4°C overnight: Anti-SNAIL + SLUG (abcam, ab180714, 1:50 dilution), Anti-IL-1R1 (clone 40101, Abnova, MAB12554, 1:50 dilution), anti-vWF (abcam, ab11713, 1:100 dilution).

Mouse ApoE^−/−^ species (Jackson Laboratory) were fed high fat diet (TD 88137, Harlan-Teklad) for 4 weeks to induce spontaneous atherosclerosis, and tissue was harvested and stored in 10% (v/v) formalin, until processing as previously shown (25). Briefly, tissue sections were dewaxed and rehydrated through graded alcohols to water. Heat-mediated antigen retrieval was performed as mentioned above and slides were cooled to room temperature before blocking in 10% horse serum/1% BSA for 30 min. Streptavidin/Biotin (Vector Labs Cat. # SP-2002) blocking was performed according to the manufacturer's recommendations. Primary antibodies: mouse Anti-IL-1R1 (R&D Systems, AF771, 0.2 mg/ml, 1:50 dilution), Anti-CD31 (Clone SZ31) (Dianova, DIA-310, 1:20 dilution) and Anti-SNAIL + SLUG (abcam, ab180714, 1:50 dilution) were incubated overnight at 4°C.

Slides were incubated with secondary antibodies after frequent washing with 0.01% (v/v) TBST for 1 h (1:2,000 dilution each; Donkey anti-mouse Alexa Fluor 555—A32773, Donkey anti-mouse Alexa Fluor 488—A32766tr, Donkey anti-rabbit Alexa Fluor 488—A32790tr) or for the CD31 staining Donkey anti-rat IgG Biotin-SP (Jackson ImmunoResearch, 712-065-150, 1:100 dilution) at room temperature. All secondary antibodies were diluted in 10% horse serum/1% BSA. Slides were washed with 1× PBS three times for 5 min each, then incubated with Streptavidin DL649 (Vector Labs, SA-5649-1, 1:200 dilution in PBS) for 1 h. After frequent rinsing in PBS and washing for 5-minute three times, the slides were mounted in Prolong™ Glass Antifade Mountant containing NucBlue (P36985). H&E images were captured using Cytation 5. Immunofluorescent images were captured using an EVOS M5000 microscope (Thermofisher). Analysis was performed using Image J and the investigators were blinded during the data collection and analysis.

### 2.6. Immunocytochemistry

For cellular immunostaining, the protocol used was adapted from (28) with slight modifications. Briefly, the cells were fixed in 3.7% (v/v) paraformaldehyde for 30 min followed by frequent rinsing in 1× PBS. The permeabilization was performed using 0.01% Triton-X100 in PBS for 10 min, and the slides were blocked in 10% horse serum/1% BSA solution for 1 h. at room temperature. Cells were incubated with primary antibodies at 4°C overnight and the primary antibodies used were anti-CD31 (abcam, ab9498, 1:100 dilution), and anti-N cadherin (abcam, ab18203, 1:100 dilution). After subsequent washing in 0.01% v/v TBST, the cells were incubated with Alexa Fluor 488 phalloidin (A12379, 1:400 dilution) mixed in 10% horse serum/1% BSA solution with secondary antibodies (1:1,000 dilution each; Donkey anti-mouse Alexa Fluor 555—A32773, Donkey anti-rabbit Alexa Fluor 647—A31573) at room temperature for 1 h. Cells were rinsed thoroughly with 1× PBS and then slides were mounted in Prolong™ Glass Antifade Mountant containing NucBlue (P36985). Images were acquired using an EVOS M5000 microscope.

### 2.7. Real-Time PCR

mRNA was extracted and isolated using RNeasy Mini Kit (Qiagen, Cat. # 74104) according to the manufacturer's instructions. Reverse transcription and real-time PCR were conducted as previously demonstrated (25). Ct values of the target genes were normalized upon the Ct values of the housekeeping genes (RPL13A and GAPDH). All PCR analysis was conducted in duplicates and the data are from 4 independent experiments. Primer sequences for the human target genes used as following: SNA1 Forward (CTCTTTCCTCGTCAGGAAGC), SNA1 reverse (CGGTGGGGTTGAGGATCT), GAPDH Forward (GAAGGTGAAGGTCGGAGTC), GAPDH Reverse (GAAGATGGTGATGGGATTTC), RPL13A Forward (GCCATCGTGGCTAAACAGGTA), RPL13A Reverse (GTTGGTGTTCATCCGCTTGC), Vimentin Forward (AGTCCACTGAGTACCGGAGAC), Vimentin Reverse (CATTTCACGCATCTGGCGTTC), SMA Forward (GCGTGTAGCACCTGAAGAG), SMA Reverse (GAATGGCGACGTACATGGCA), VECAD Forward (CAGCCCAAAGTGTGTGAGAA), VECAD Reverse (TGTGATGTTGGCCGTGTTAT), CD31 Forward (GAGTCCAGCCGCATATCC), CD31 Reverse (TGACACAATCGTATCTTCCTTC). Mouse target genes as following: mGAPDH Forward (CTTCACCACCTTCTTGATGTC), mGAPDH Reverse (CTTCACCACCTTCTTGATGTC), mB2MG Forward (CAGTCGCGGTCGCTTCAGTC), mB2MG Reverse (CAGTATGTTCGGCTTCCCATTC), mSnai1 Forward (CTTGTGTCTGCACGACCTGT), mSnai1 Reverse (CTTCACATCCGAGTGGGTTT), mCD31 Forward (GGAGTCAGAACCCATCAGGA), mCD31 Reverse (CAGCTGGTCCCCTTCTATGA), mIl1r1 Forward (GAATGACCCTGGCTTGTGTT), mIl1r1 Reverse (TGTGCTCTTCAGCCACATTC).

### 2.8. Bioinformatics analysis

All bioinformatics analysis were performed in R. The RNA microarray data was processed by “Limma” R package (30) and visualized by “EnhancedVolcano” (31) and “pheatmap” (32). Single cell RNA seq data was analyzed by “Seurat” (33).

### 2.9. Statistical analysis

Data is analyzed as mean ± standard error of the mean (SEM) using GraphPad prism software (Version 9.5, GraphPad, San Diego, CA). For multiple comparisons, data were analyzed by 1-Way ANOVA followed by Tukey's post-test to compare data with one independent variable, whereas for the multiple comparisons of data with two independent variables we used 2-Way ANOVA and Bonferroni's post-test. An unpaired student's *t*-test was used to compare two different datasets. Statistical significance was achieved when *p* < 0.05.

## 3. Results

### 3.1. IL-1R1 expression is associated with EndMT in human unstable atheroma

**Figure 1:**
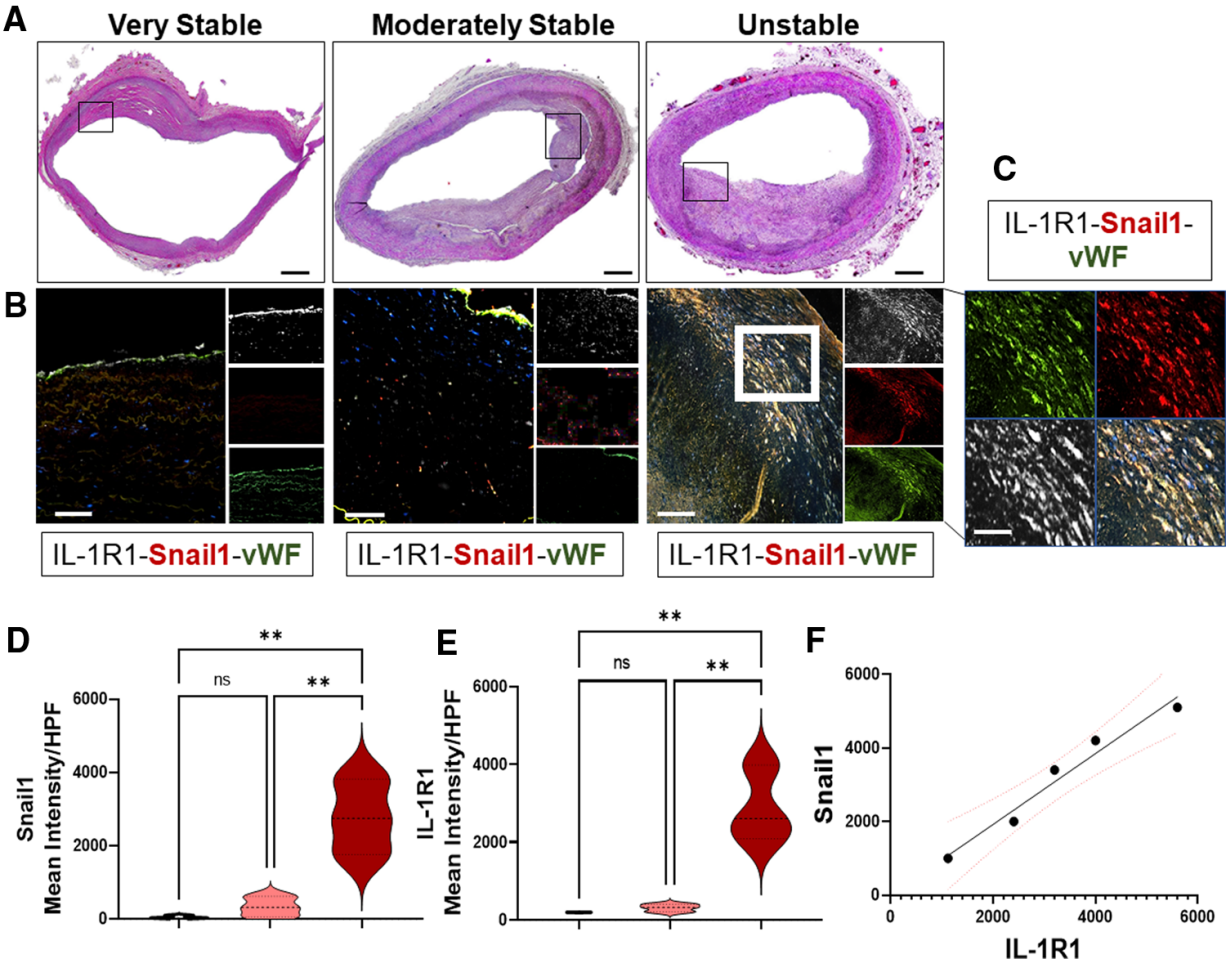
EndMT in human unstable atheroma is associated with IL-1R1 expression. (**A**) Sections of human coronary atherosclerosis graded into very stable, moderately stable, and unstable lesions according to AHA recommendations and stained with H&E, scale bars = 1,000 µm, black boxes indicate the areas of interest that were stained for immunofluorescence and captured in B. (**B,C**) Human lesions were stained for IL-1R1 (white), vWF (green) and Snail1 (red) and showing increase expressions between these proteins within unstable lesions. Scale bars = 100 µm, white box indicates the higher magnification area demonstrated in (**C**), scale bar in C indicates 50 µm. (**D,E**) Semi-quantification to the expression levels of IL-1R1 and Snail1 (Mean Intensity) per high power field (HPF) and F is an analytic correlation between Snail1 and IL-1R1 expressions in unstable lesions, red dots indicate the 95% Confidence Intervals, Pearson *r* = 0.9808, *p* (two tailed) = 0.0032. Data are from *n* = 5 different human samples, and are mean ± SEM, analyzed by 1-way ANOVA and Tukey's post-test, **p < 0.01, ns, non-significant.

Previously we and others have shown that IL-1 in human coronary atherosclerotic plaques is predominantly expressed within the endothelium (22–24), demonstrating a role for endothelial IL-1 signaling in the pathogenesis of atherosclerosis. However, the data on its receptor, the IL-1R1, its site of expression in human atherosclerotic plaques are still unclear. Therefore, we analyzed sections of human coronary atherosclerotic lesions to detect the cell type-specific expressing IL-1R1. To further explore whether the expression of IL-1R1 is altered by the advanced stages of atherosclerosis, we categorized the human progressive atherosclerotic plaques into two grades, either IV or V as per the AHA recommendations (34). These lesions were further categorized into very stable, moderately stable, and unstable lesions depending on the stability characteristics that are established by (1) using extensive histological landmarks and blindly assessed and graded by a pathologist at Louisiana State University Health Sciences Center at Shreveport (Figure 1A).

To investigate IL-1R1 expression between the three phenotypically different stages of human atheroma, the lesions were stained for IL-1R1, Snail1 (EndMT transcription factor), and von-Willebrand factor: vWF (Endothelial marker) (Figures 1B–E). Interestingly, lesions that are very stable show predominant expression of IL-1R1 within the endothelium and hardly any Snail1 expression, suggesting that EndMT is not happening in the stable human atherosclerosis (Figure 1B). Surprisingly, we observed an increased expression of IL-1R1 mainly in a sub-endothelial population that are co-expressing Snail1 and vWF (Figures 1C–E). The increase in Snail1and IL-1R1 proteins were significantly higher in unstable lesions compared to moderately stable lesions (Figures 1D,E) Furthermore the association between IL-1R1 and Snail1 expressions in unstable lesions was very significant (*p* = 0.0032) (Figure 1F). Collectively, the data demonstrates that IL-1R1 is expressed predominantly in endothelial cells in stable advanced human atherosclerotic plaques, however, the expression of IL-1R1 is increased in EndMT in unstable human atherosclerosis.

To confirm the causal association between IL-1R1 and EndMT in human unstable atherosclerosis, we first re-assessed the relative expression of active IL-1R1 and EndMT genes in macroscopically intact tissue vs. atheroma tissue from an online dataset (GSE43292) (35). The selected dataset was used because it provides a direct assessment to the list of genes that are upregulated in the intimal area of human unstable lesions and compared them with a macroscopically intact area from the same patient. In the original study, the endarterectomy specimens were characterized histologically according to the AHA and most atheroma samples were presented at stage IV and V lesions, and the macroscopically considered as “intact tissue” was almost exclusively composed of stage I and II lesions (35).

**Figure 2:**
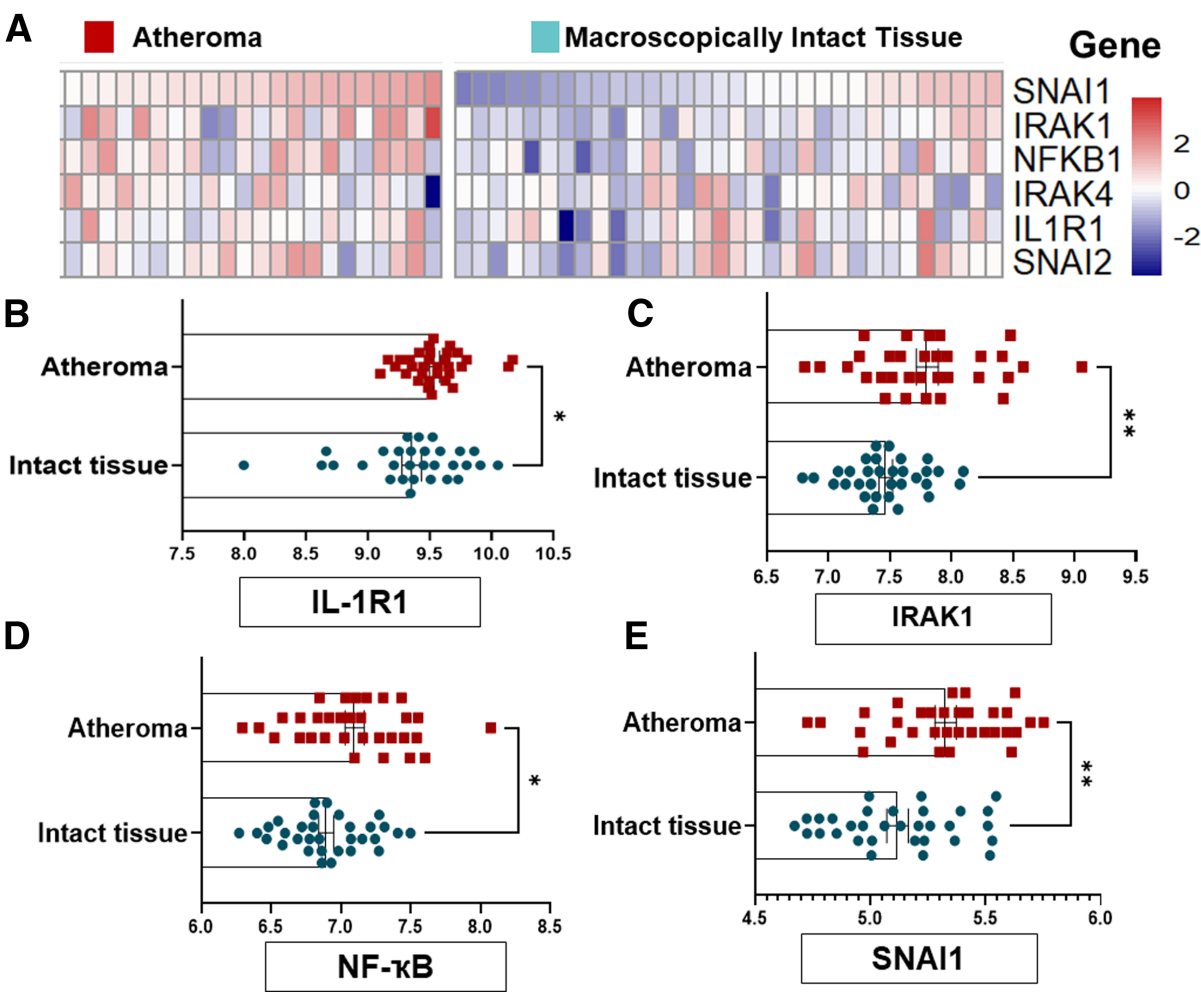
EndMT and IL-1R1 complex are highly expressed in human atheromatous lesions compared to intact tissue. (**A**) GSE43292 dataset was analyzed and (**B**) the heat map to SNAI1, IRAK1, NF-kB, IRAK4, IL-1R1 and SNAI2 was shown. (**B–E**) expression levels of the IL-1R, IRAK1, NFKB1 and SNAI1 genes from *n* = 32 individuals per group, the value (intensity) of each probe represents the RNA expression level. Data was analyzed as mean ± SEM, and statistical analysis was conducted by unpaired *t* test, *p < 0.05, **p < 0.01.

The groups of genes that are differentially expressed between either the macroscopically intact (areas surrounding the atheroma from the same patient samples) or atheroma tissue (from carotid endarterectomy samples) of *n* = 32 human subjects each were re-analyzed for genes that are involved in IL-1R1 activation mainly IRAK1/4 (Interleukin-1 Receptor Associated Kinase 1/4) and NFKB1 (nuclear factor kappa B1), and EndMT transcriptional factors SNAI1 (Snail1) and SNAI2 (Snail2), which were further blotted using a heat map (Figure 2A).

Interestingly, we examined the relative expression IL-1R1, IRAK1, NFKB and SNAI1) and found a significant differences in these genes' levels with a marked increase in the atheroma compared to intact tissues (Figures 2B–E). Collectively, these findings suggest that the EndMT and active IL-1R1 are highly expressed in human severe atherosclerotic lesions as compared to intact tissues, and may suggest that the regional expressions of IL-1R1 and EndMT is induced by local factors rather than systemic effects.

### 3.2. IL-1R1 expression is associated with EndMT under areas of disturbed blood flow

**Figure 3:**
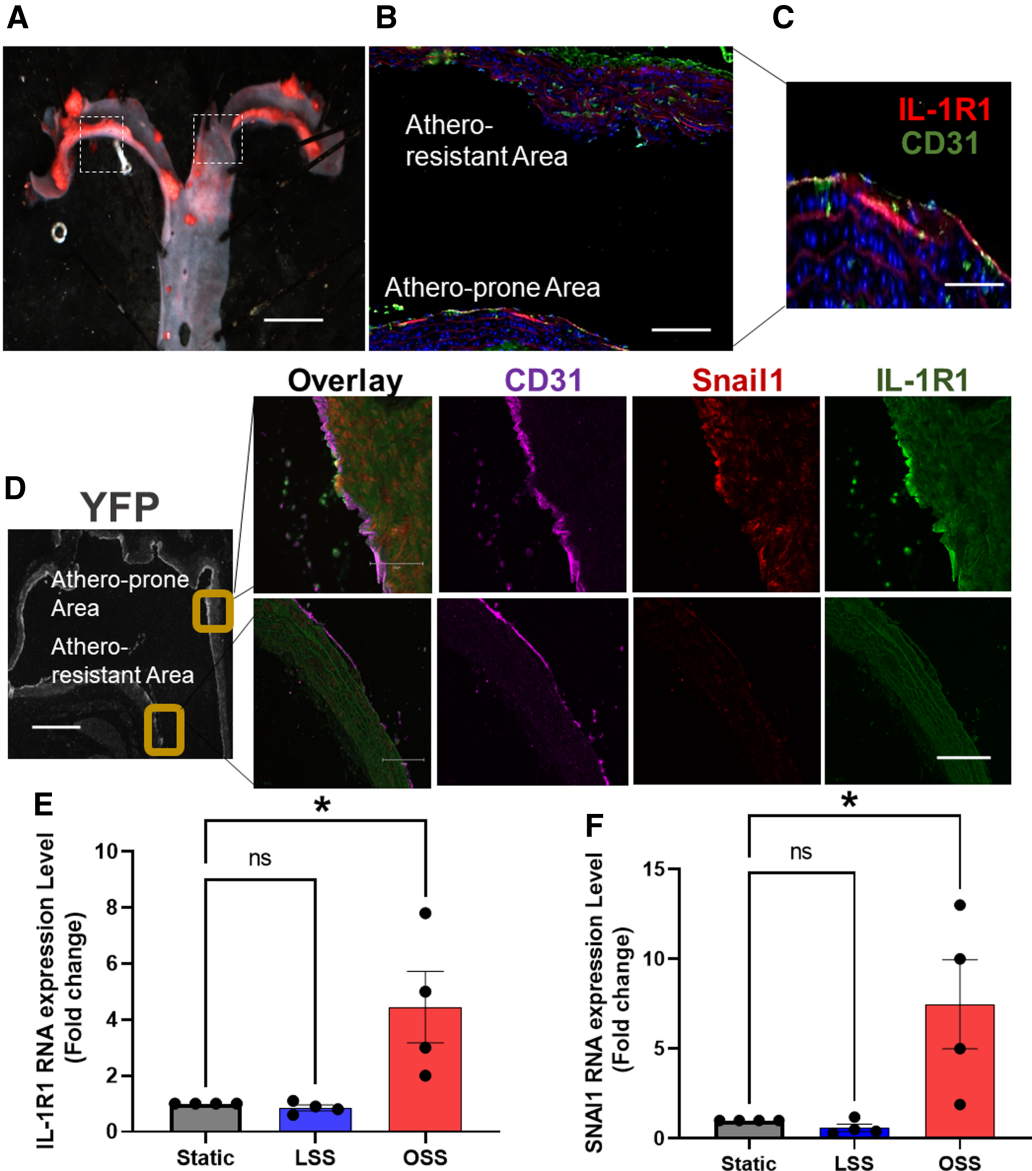
IL-1R1 expression is associated with EndMT under disturbed blood flow areas. (**A**) Representative image of Oil O Red-stained aortic arch section of ApoE KO. Scale bars = 1 mm. Dash boxes represent the athero-resistant and atheroprone areas. (**B,C**) Immunofluorescent staining of IL-1R1 (red) and CD31 (green). Scale bars = 50–100 µm. (**D**) Shows immunofluorescence staining of CD31 (purple) Snail (red) and IL-1R1 (green) in atheroprone and athero-resistant areas in the EC-specific lineage tracing mice, YFP positive expression in ECs as stained by GFP (grey) (*n* = 3). Scale bars = 500 − 100 µm. (**E,F**) Human aortic ECs (HAOECs) exposed to laminar shear stress (LSS 10 dynes/cm^2^; mimicking *in vivo* athero-protective flow and oscillatory shear stress (OSS; 1 dynes/cm^2^; mimicking *in vitro* atheroprone flow) for 24 h. and lysed and mRNA was quantified using qRT-PCR for IL-1R1 (**E**) and SNAI1 (**F**). Data are represented as mean ± SEM, and 1-way ANOVA and Tukey's post-test test, *p < 0.05, *n* = 4 independent experiments.

Atherosclerosis develops at sites of local endothelial cell (EC) activation, where a shift in endothelial cell phenotype in response to d-flow occurs (6, 36). The molecular mechanism by which ECs switch their phenotypes under d-flow areas is incompletely understood. D-flow (a low amplitude and a multi-directional force) (37) is present in certain parts of the arterial tree, such as the lesser curvature of the aortic arch, whereas the athero-protective high amplitude and unidirectional (laminar) flow is present in other areas, such as the greater curvature of aortic arch (38) (Figures 3A–D). EndMT has been shown to be induced by d-flow at the atheroprone areas (36, 39). Having shown that IL-1R1 expression is locally increased in human unstable atherosclerosis and that is associated with EndMT, we first asked if IL-1R1 is expressed under areas of d-flow, and if that is associated with EndMT. As expected, in mice with an atherosclerosis phenotype, IL-1R1 expression is predominant in the atheroprone areas as assessed by immunofluorescence and no IL-1R1 staining was detected in the athero-protective area of the aortic arch (Figures 3B,C).

To establish the relationship between EndMT and IL-1R1 expression we stained the aortic arch of our EC-specific lineage tracing mice (Figure 3D). The endothelial specific expression of IL-1R1 under d-flow areas was confirmed by a co-localization with CD31 and that coincides with Snail1 expression, only in the atheroprone areas (Figure 3D), confirming the association between IL-1R1 and d-flow-induced EndMT. Furthermore, we examined the effect of laminar (LSS) and oscillatory shear stress (OSS) on IL-1R1 and SNAI1 mRNA expressions *in vitro* in HAOECs. Compared to static condition, LSS did not significantly alter the mRNA levels of IL-1R1 nor SNAI1 (Snail1) in our *in vitro* flow system. Interestingly, OSS significantly increased IL-1R1 and SNAI1 mRNA expressions compared to static conditions (Figures 3E,F). We confirmed the changes in IL-1R1 at protein levels *in vitro* and relative to the athero-protective LSS, the atheroprone OSS significantly increased IL-1R1 protein expression with *p* < 0.01 (Supplementary Figure S1).

These findings further demonstrate that IL-1R1/Snail1 are induced by OSS and supports the potential role that IL-1R1 might play in d-flow-induced EndMT.

### 3.3. IL-1R1 knockdown prevents OSS-induced EndMT *in vitro*

**Figure 4:**
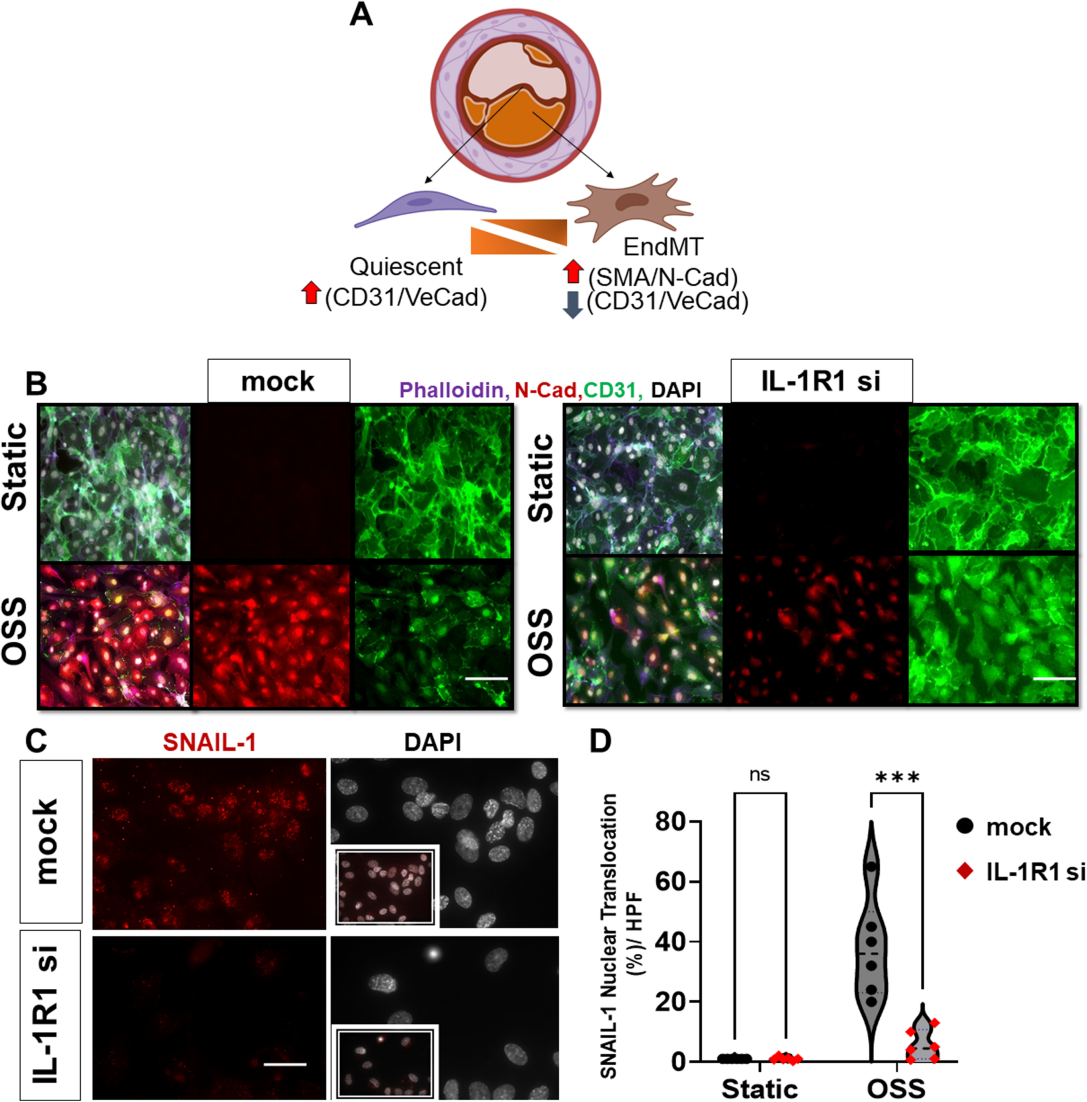
IL-1R1 knockdown prevents OSS-induced EndMT *in vitro*. (**A**) Module of Endothelial phenotypic switches to EndMT in atherosclerosis. (**B**) immunofluorescent images of mock vs. IL-1R1 siRNA HAECs subjected to oscillatory shear (OSS; 1 dynes/cm^2^ for 24 h), from *n* = 3. The cells were stained for Phalloidin (F-actin, purple), N-cadherin (red; mesenchymal marker), and CD31 (green; endothelial marker). (**C,D**) Immunofluorescent images of Snail1 (red) and DAPI (white: shows nuclei) in mock vs. IL- 1R1 siRNA HAOECs subjected to oscillatory shear (OSS; 24 h) and assessed for Snail1 nuclear translocation. Snail1 nuclear translocation was compared between IL-1R1 siRNA cells and mock controls in static and OSS conditions. Data are mean ± SEM, multiple unpaired *t* test, ***p < 0.001, *n* = 6 independent experiments. Scale bars = 400 − 100 µm.

Recent studies have demonstrated that d-flow promotes EndMT formation (36, 40, 41). However, the signaling intermediaries that link the mechanical d-flow to EndMT are still unclear. During EndMT, endothelial cells express mesenchymal markers such as N-cadherin, alpha smooth muscle actin (α-SMA), vimentin, in addition to the induction of the transcriptional factor Snail1. These mesenchymal markers drive the phenotypic changes in ECs that involve loss of EC markers such as VE-cadherin and CD31 by a Snail1 gene upregulation, and subsequent loss of cell-cell contacts (Figure 4A) (8, 42–44). To determine if IL-1R1 is involved in induction of d-flow-induced EndMT, we first examined silencing of IL-1R1 using SMARTpool siRNA to IL-1R1 (IL-1R1 siRNA) *in vitro* in HAOECs and then the cells were subjected to OSS using the Ibidi flow system for 24 h (Figures 4B–D). EndMT induction by the athero-prone OSS was assessed using a combination of immunofluorescent staining. Compared to mock control, IL-1R1 siRNA treated cells showed a remarkable reduction in N-cadherin (mesenchymal cell marker) and an increase in the endothelial CD31 (endothelial cell marker) expression after OSS exposure (Figure 4B). Consistent with this, OSS increased Snail1 nuclear translocation in mock cells, which was significantly decreased in IL-1R1-deleted ECs (Figures 4C,D).

**Figure 5:**
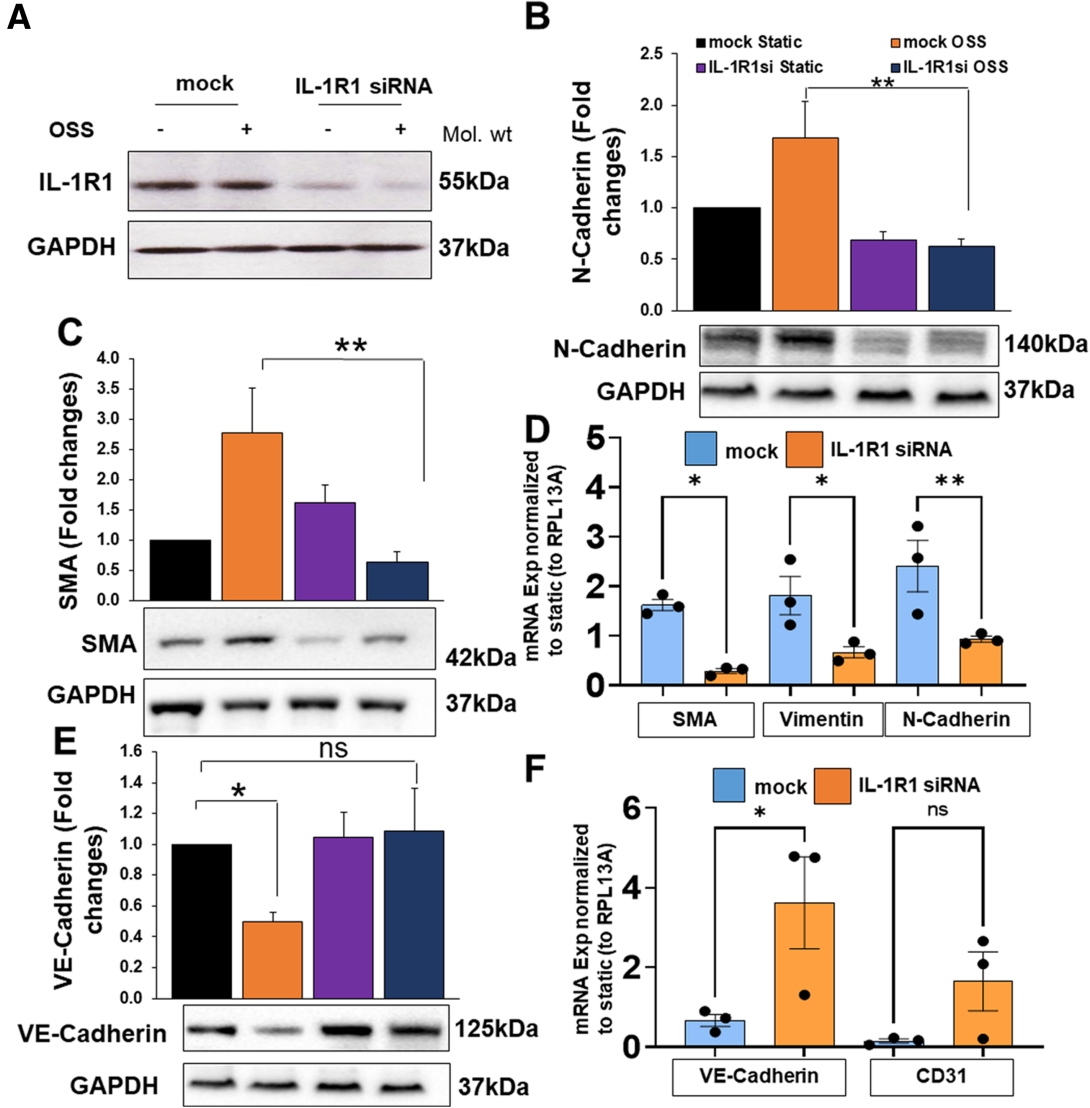
HAOECs were transfected with siRNA targeting IL-1R1 or mock and exposed to oscillatory shear stress (OSS) for 24 h. Cells lysates were collected for proteins of IL-1R1 (**A**), N-Cadherin (**B**), α-SMA (**C**), or mRNA (**D**). Protein or mRNA levels of endothelial markers Ve-Cadherin (**E**), and CD31 (**F**) were quantified using Western blot or qRT-PCR. Data analyzed by 2-way ANOVA and Bonferroni's post-test; *p < 0.05, **p < 0.01, representative blots are from *n* = 4–5 independent experiments.

To assess the protein and mRNA levels of endothelial and EndMT markers by OSS, we measured proteins using Western blot and mRNA levels using qRT-PCR (Figure 5) in mock vs. IL-1R1 deleted ECs. The transfection efficiency of IL-1R1 siRNA in HAOECs was confirmed (Figure 5A). Consistent with our immunofluorescent staining, OSS induced an upregulation in N-cadherin and α-SMA protein and mRNA levels (Figures 5B–D) and vimentin mRNA expression (Figure 5D) in the mock control, whereas IL-1R1 siRNA pre-treatment in ECs showed a significant decrease in the induction of these markers even after the OSS exposure (Figures 5B–D). Furthermore, OSS induced a downregulation in endothelial markers, which was prevented in IL-1R1 siRNA cells (Figures 5E,F). Likewise, in mouse IL-1R1 KO ECs that were exposed to OSS, the induction in the EndMT marker was remarkably decreased compared to WT cells (Supplementary Figure S2).

Collectively, the data suggests that IL-1R1 is implicated in an upstream signaling to the mesenchymal phenotype in HAOECs when exposed to the athero-prone oscillatory flow.

### 3.4. IL-1R1 knockdown prevents OSS-induced EndMT *in vivo*

**Figure 6:**
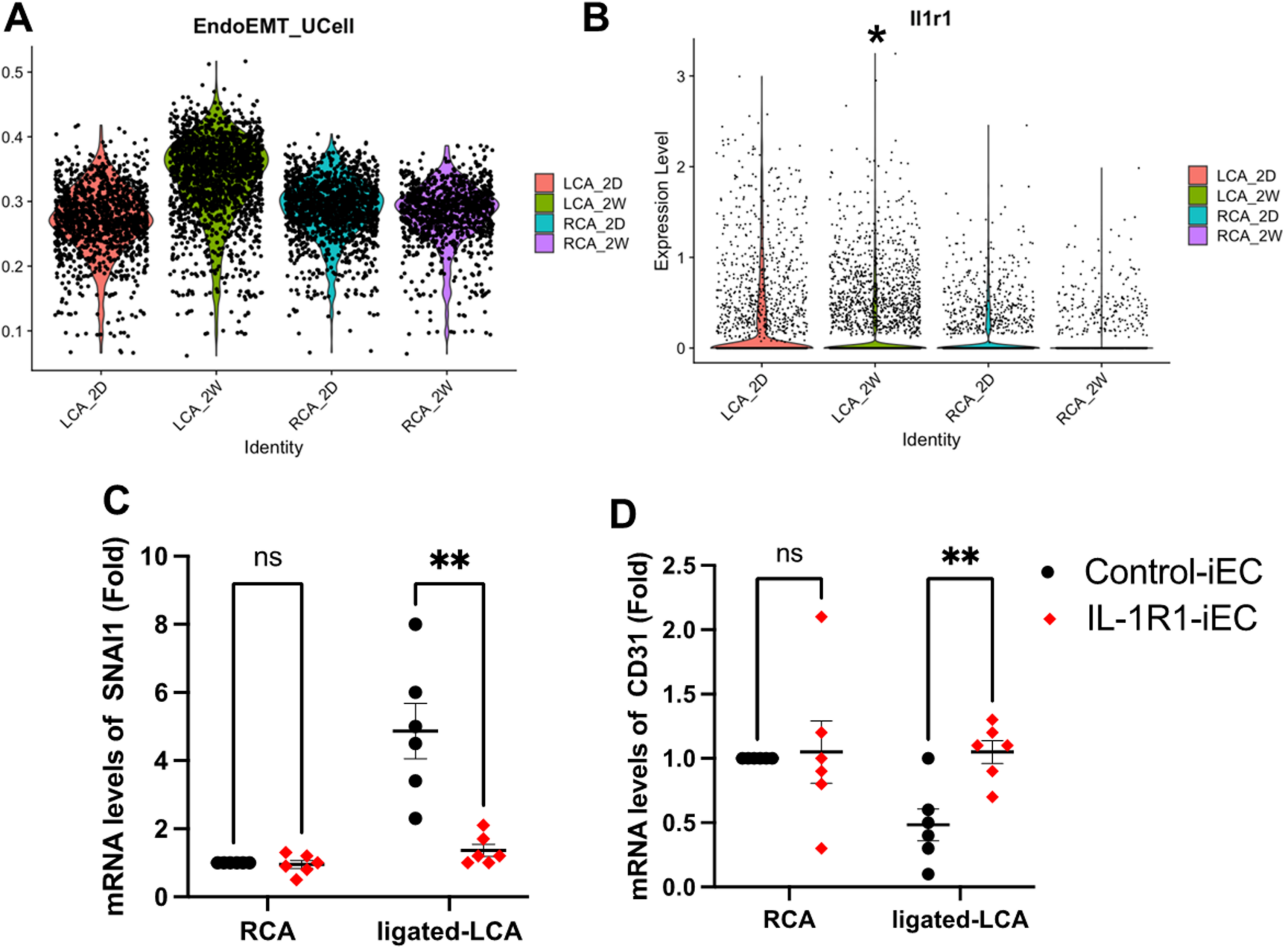
IL-1R1 knockout prevents d-flow-induced EndMT *in vivo*. (**A,B**) sc-RNA seq datasets (Bioproject #PRJNA646233) are re-analyzed. (**A**) EndMT clusters were induced by d-flow after 2 weeks of left carotid artery ligation (LCA) maximum after 2 weeks (LCA_2W). (**B**) IL-1R1 (Il1r1) expression levels in left carotid artery (LCA) compared to right carotid artery (RCA) 2 days (2D) and 2 weeks (2W) post-ligation. (**C**) Intimal mRNA levels of SNAI1 and CD31 (**D**) were compared 2 weeks after ligation between Control-iEC and IL-1R1-iEC mice. Data are mean ± SEM, analyzed by multiple unpaired *t* test, *n* = 6 mice per condition, *p < 0.05, **p < 0.01.

To confirm that IL-1R1 is induced by d-flow *in vivo* and that this induction is associated with EndMT, we re-analyzed an online dataset (Bioproject #PRJNA646233) from EC-enriched single cell RNA seq analysis after a mouse partial carotid ligation surgery (6). The sc-RNA seq was comprised of transcript expression results of left carotid artery (LCA: exposed to the atheroprone d-flow) and right carotid artery (RCA: exposed to the athero-protective laminar flow) obtained at 2 days and 2 weeks post-ligation. We first re-blotted the EndMT population and compared their induction levels under different time points in both RCA and LCA (Figure 6A). Our re-analysis confirmed the induction of EndMT cells in the ligated LCA compared to the unligated RCA after 2 weeks. Next, we re-tested the differential expression of IL-1R1 in the ligated LCA and un-ligated RCA among the EndMT population between 2 days and 2 weeks. Interestingly, we observed a significant increase in IL-1R1 expression in the ligated LCA (d-flow induced) compared to un-ligated RCA (laminar flow) after 2 weeks (Figure 6B).

To determine if IL-1R1 directly contributes to EndMT induction by d-flow *in vivo*, we used our own mouse model of Control-iEC (VE-cadherin-CreERT2^tg/+^, ApoE^−/−^) and IL-1R1^iEC−KO^ (VE-cadherin-CreERT2^tg/+^, IL-1R1^flox/flox^, ApoE^−/−^) mice. After tamoxifen injection, we confirmed the induced IL-1R1 downregulation within the intima of the IL-1R1^iEC−KO^ mice compared to Control-iEC (Supplementary Figure S3). The experimental groups, Control-iEC and IL-1R1^iEC−KO^ were subjected to the partial carotid ligation surgery to induce d-flow in the LCA. EndMT gene SNAI1 and endothelial CD31 were measured following isolation of intimal mRNA by qRT-PCR after 2 weeks post-ligation (Figures 6C,D). Consistent with our *in vitro* findings, the intimal mRNA levels of SNAI1 were remarkably increased by d-flow following the ligation of LCA in the control mice and that was significantly decreased in IL-1R1^iEC−KO^ mice (Figure 6C). By contrast, d-flow induction in the ligated LCA reduced CD31 mRNA levels in control animals and that was significantly increased in IL-1R1^iEC−KO^ (Figure 6D). Collectively, these results demonstrate that IL-1R1 inhibition in mice prevents d-flow-induced EndMT and maintain the EC phenotype.

## 4. Discussion

Accumulative evidence has demonstrated the phenotypic transition of endothelium to EndMT in human atheroma, and new emerging evidence with the single cell RNA studies have been reported on the presence of EndMT in mouse atherosclerotic plaques (43–45). D-flow is one of the main triggers to EndMT formation (36, 39), however, there is little evidence on the signaling intermediaries that link d-flow to EndMT formation. Herein, we described a novel role for endothelial IL-1R1 in d-flow-induced EndMT *in vitro* and *in vivo*. We found for the first time, that IL-1R1 is predominantly expressed in endothelium of human coronary stable atherosclerotic plaques. The endothelial expression of IL-1R1 is increased in unstable lesions and is associated with the EndMT transcriptional factor Snail1. In our endothelial cell-specific lineage tracing mice, we observed the predominant expression of IL-1R1 and Snail1 in areas exposed to d-flow. Inhibition of IL-1R1 resulted in down-regulation of EndMT markers after OSS exposure *in vitro*. Importantly, in our endothelial cell-specific IL-1R1 KO mice we confirmed the direct effect of IL-1R1 inhibition on d-flow-induced EndMT in the partial carotid ligation model.

Endothelial-to-mesenchymal transition (EndMT) is a process whereby endothelial cells undergo a series of molecular events that lead to phenotypic changes towards mesenchymal cells (40). EndMT plays a fundamental role during development (46) but there is a mounting evidence now that suggests EndMT is also involved in atherosclerosis (36, 39, 43). EndMT is characterized by multiple morphological and physiological changes, including loss of endothelial cell polarity, and disruption of intercellular junctions (42), so they can migrate in plaques. EndMT has been identified in progressive atherosclerotic plaques, and the extent of EndMT observed in the human plaques strongly correlates with the severity of the disease (43), implying clinical relevance of EndMT in the pathogenesis of atherosclerosis. However, the field faces several uncertainties regarding at which stage of human atherosclerosis EndMT appears to occur, and how that contributes to lesion stability, especially in the advanced stages where patients presented in the clinic with the symptoms of acute coronary syndrome. Our data using a blinded staging system and following the AHA recommendations for plaque characteristics (1, 2) detected EndMT in unstable stages IV and V human coronary atherosclerosis, whereas we did not observe EndMT in the stable stages. This finding is important as it implies that EndMT is involved in plaque stability, however, further characterization studies on their roles are warranted.

Interleukin-1 (IL-1) refers to two closely related cytokines IL-1α and IL-1β (47). Of the two, the leaderless cytokine IL-1β has been extensively investigated, and it has been shown to be involved in every stage of atherosclerosis from early lesion formation to progressive late-stage lesions (48). Global targeting of IL-1β demonstrated a decrease in atherosclerosis burden in mice (49, 50). Subsequently, the major clinical trial of inhibiting IL-1β with an antibody, canakinumab, administered in patients with myocardial infarction was concluded in 2017 showing a major reduction in cardiovascular mortality in sub-sets of patients. In this study, the magnitude of inflammation achieved in patients with high IL-6 and CRP was directly related to the magnitude of the clinical benefits of using anti-IL-1 (51), confirming the role of inflammation in CAD. However, some of those patients developed immunosuppressive off-target effects (11), which could be mitigated with an antibiotic regime (52). While in atherosclerosis it could be beneficial to inhibit immune cell infiltrations as evident that treatment with anti-IL-1B can provide additional cardio-protective benefits by reducing leukocyte recruitment to the atherosclerotic plaque as well as reducing its size (53). Nevertheless, the off-target effects on innate immune response maybe due to the systemic rather than local inhibition of IL-1.

Intriguingly, two different research groups showed that inhibiting of IL-1β signaling in vascular wall rather than systemic inhibition is responsible for the beneficial effect on atherosclerosis burden reduction. Global IL-1R1 deletion in mice had significantly less atheroma and seems to not be altered in bone marrow transfer experiments (19), suggesting that vascular wall inhibition rather than myeloid inhibition is responsible (54). However, within the vascular wall, the data on IL-1R1 are still conflicting as to which cell-type specific IL-1R1 could be inhibited. Smooth muscle specific deletion of IL-1R1 showed high proinflammation and more instability features, whereas macrophage-specific deletion of IL-1R1 demonstrated less inflammation and more stable characteristics (20). In patients, there is no clear evidence on which one of the vascular wall cells is responsible for IL-1R1 effects, and whether the signaling through the receptor is different in different cell-types. Our study is the first to demonstrate an enhanced IL-1R1 expression in human atherosclerosis stages IV and V, and that is predominantly within the endothelium. While multiple studies on IL-1R1 have been diverse and even controversial, our study found that IL-1R1 expression is increased in the endothelium and that coincides with the appearance of EndMT in unstable lesions. These results align with the previously reported findings illustrating the predominate expression of IL-1β within the endothelium in human coronary atherosclerosis (24, 29), suggesting the critical signaling of IL-1 within the endothelium.

Moreover, we found that the IL-1R1 expression is enhanced under d-flow areas. Both disturbed blood flow (d-flow) and IL-1 have been implicated in EndMT (36, 44). The signaling pathway that links d-flow and EndMT is still unclear. Our recent work has demonstrated that d-flow induces interleukin-1 receptor signaling kinase within human and mouse progressive atherosclerosis (25). Interestingly, our current study determines the dynamic interplay between disturbed flow and IL-1 signaling in EndMT, as we showed that deletion of IL-1R1 *in vitro* and *in vivo* significantly prevented d-flow-induced EndMT. EndMT is a spectrum of phenotypic changes in ECs and whether inhibition of IL-1R1 mediated EndMT in atherosclerosis is yet to be investigated and future studies from our laboratory will focus on dissecting the downstream pathway(s) involved.

### 4.1. Limitation of the study

Our study provides the first assessment to the mechanical response of endothelial IL-1R1 to disturbed flow and EndMT formation *in vitro* and *in vivo*. Our study has strengths as well as some limitations. Firstly, we performed experiments on ApoE KO mice to study endothelial-specific IL-1R1 deletion on disturbed flow-induced EndMT. Our first observations on the association between IL-1R1 and Snail1 in human unstable atherosclerosis where the lesions exhibit more complex and advanced phenotype than that to the mice. The use of mice to study human pathology has some inherent limitations, which should be considered before any conclusions can be drawn. Mice do not usually develop atherosclerosis, due to the high protective HDL-C in their plasma, therefore, by knocking out ApoE and feeding the mice on a high fat diet for 4 weeks, we assessed the earliest possible changes in the vasculatures. We are not expecting the lesions that are developed in our mouse model to be phenotypically similar to that in humans, especially with the advanced stage IV and V lesions. This could explain why the staining pattern in mice is substantially different than that in humans. Secondly, we assessed the mRNA expressions of Snail1, CD31, and IL-1R1 in the intimal samples of ligated LCA and un-ligated RCA after the partial carotid ligation surgery in the Control-iEC and IL-1R1^iEC−KO^ animals. It could be there is some contamination in the samples with the medial mRNAs. However, we used a standard method by TRIzol flush after tissue harvesting, and tested the purity of the isolation as established by our research group (25, 28) and others (6, 55).

### 4.2. Clinical significance of the current study

Coronary artery disease (CAD) accounts for ∼600,000 deaths/year in the USA, contributing to significant morbidities (56). Atherosclerosis, an underlying disease process of CAD, is characterized by abnormal accumulation of lipids and proinflammatory cells within the vascular wall (57) which can rupture, leading to sudden death or acute myocardial infarction (58). While current treatment strategies, including percutaneous coronary intervention, are used to improve the quality of life, they fail in preventing the severity of disease (59), leading to an urgent need for uncovering novel and specific therapeutic targets to prevent atherosclerosis-induced mortality. Early clinical observations suggest that atherosclerosis occurs at the arterial sites of disturbed blood flow (d-flow) (60). In response to d-flow, endothelial cells (ECs) acquire a mesenchymal phenotype through endothelial-to-mesenchymal transition (EndMT) (36, 40). EndMT is implicated in the progression of atherosclerosis, where it leads to plaque destabilization by promoting local inflammation (8). Interleukin-1 (IL-1) is highly implicated in atherosclerosis progression (19–21, 61) and development of EndMT (16). The therapeutic approach of blocking IL-1 using an antibody, Canakinumab, significantly reduced mortality rates in CAD patients (12, 62) but it also induced systemic off-target effects due to low circulating leukocyte counts (12), limiting the global targeting of IL-1. Thus, although inhibition of IL-1 is a great strategy to prevent progression of atherosclerosis, we need novel therapeutic targets for specific inhibition of IL-1 within the vascular wall to avoid systemic off-target effects on immune cells (47).

In conclusion, global inhibition of IL-1 in patients with underlying atherosclerosis has already been tested in the completed CANTOS study with some promising effects (11). However, the data on interleukin-1 receptor IL-1R1 in mice are still conflicting (20, 21). Herein with our results, we showed that endothelial IL-1R1 expression is implicated in unstable atheroma undergoing EndMT. Furthermore, our study illustrates a crosstalk between d-flow and IL-1R1 in endothelial cells and demonstrates a novel role for the endothelial IL-1R1 expression in EndMT formation after d-flow exposure, which represents a new direction for potential precision therapy by targeting IL-1R1 within the endothelium. This might lead to better strategies in managing progressive atherosclerosis and maintaining plaque stability in patients.

## Data Availability

The original contributions presented in the study are included in the article/**Supplementary Material**, further inquiries can be directed to the corresponding author.

## References

[1] Virmani R Kolodgie FD Burke AP Farb A Schwartz SM. Lessons from sudden coronary death: a comprehensive morphological classification scheme for atherosclerotic lesions. Arterioscler Thromb Vasc Biol. (2000) 20:1262–75. 10.1161/01.atv.20.5.1262 10807742

[2] Kolodgie FD Virmani R Burke AP Farb A Weber DK Kutys R Pathologic assessment of the vulnerable human coronary plaque. Heart. (2004) 90:1385–91. 10.1136/hrt.2004.041798 15547008 PMC1768577

[3] Orekhov AN Andreeva ER Krushinsky AV Novikov ID Tertov VV Nestaiko GV Intimal cells and atherosclerosis. Relationship between the number of intimal cells and major manifestations of atherosclerosis in the human aorta. Am J Pathol. (1986) 125:402–15. 3789095 PMC1888230

[4] Shankman LS Gomez D Cherepanova OA Salmon M Alencar GF Haskins RM KLF4-dependent Phenotypic modulation of smooth muscle cells has a key role in atherosclerotic plaque pathogenesis. Nat Med. (2015) 21:628–37. 10.1038/nm.3866 25985364 PMC4552085

[5] Evrard SM Lecce L Michelis KC Nomura-Kitabayashi A Pandey G Purushothaman KR Endothelial to mesenchymal transition is common in atherosclerotic lesions and is associated with plaque instability. Nat Commun. (2016) 7:11853. 10.1038/ncomms11853 27340017 PMC4931033

[6] Andueza A Kumar S Kim J Kang DW Mumme HL Perez JI Endothelial reprogramming by disturbed flow revealed by single-cell RNA and chromatin accessibility study. Cell Rep. (2020) 33:108491. 10.1016/j.celrep.2020.108491 33326796 PMC7801938

[7] Alencar GF Owsiany KM Karnewar S Sukhavasi K Mocci G Nguyen AT Stem cell pluripotency genes Klf4 and Oct4 regulate complex SMC phenotypic changes critical in late-stage atherosclerotic lesion pathogenesis. Circulation. (2020) 142:2045–59. 10.1161/CIRCULATIONAHA.120.046672 32674599 PMC7682794

[8] Kovacic JC Dimmeler S Harvey RP Finkel T Aikawa E Krenning G Endothelial to mesenchymal transition in cardiovascular disease: JACC state-of-the-art review. J Am Coll Cardiol. (2019) 73:190–209. 10.1016/j.jacc.2018.09.089 30654892 PMC6865825

[9] Beranek JT Cavarocchi NC. Undifferentiated vascular endothelial cells in coronary allograft atherosclerosis. Int J Cardiol. (1990) 28:127–8. 10.1016/0167-5273(90)90021-v 2242124

[10] Hirschfield GM Pepys MB. C-reactive protein and cardiovascular disease: new insights from an old molecule. QJM. (2003) 96:793–807. 10.1093/qjmed/hcg134 14566035

[11] Ridker PM Everett BM Thuren T MacFadyen JG Chang WH Ballantyne C Antiinflammatory therapy with canakinumab for atherosclerotic disease. N Engl J Med. (2017) 377:1119–31. 10.1056/NEJMoa1707914 28845751

[12] Ridker PM MacFadyen JG Everett BM Libby P Thuren T Glynn RJ Relationship of C-reactive protein reduction to cardiovascular event reduction following treatment with canakinumab: a secondary analysis from the CANTOS randomised controlled trial. Lancet. (2018) 391:319–28. 10.1016/S0140-6736(17)32814-3 29146124

[13] Sims JE Smith DE. The IL-1 family: regulators of immunity. Nat Rev Immunol. (2010) 10:89–102. 10.1038/nri2691 20081871

[14] Dinarello CA. A clinical perspective of IL-1 beta as the gatekeeper of inflammation. Eur J Immunol. (2011) 41:1203–17. 10.1002/eji.201141550 21523780

[15] Chamberlain J Evans D King A Dewberry R Dower S Crossman D Interleukin-1 beta and signaling of interleukin-1 in vascular wail and circulating cells modulates the extent of neointima formation in mice. Am J Pathol. (2006) 168:1396–403. 10.2353/ajpath.2006.051054 16565512 PMC1606552

[16] Maleszewska M Moonen JR Huijkman N van de Sluis B Krenning G Harmsen MC. IL-1beta and TGFbeta2 synergistically induce endothelial to mesenchymal transition in an NFkappaB-dependent manner. Immunobiology. (2013) 218:443–54. 10.1016/j.imbio.2012.05.026 22739237

[17] Ridker PM Howard CP Walter V Everett B Libby P Hensen J Effects of interleukin-1beta inhibition with canakinumab on hemoglobin A1c, lipids, C-reactive protein, interleukin-6, and fibrinogen: a phase IIb randomized, placebo-controlled trial. Circulation. (2012) 126:2739–48. 10.1161/CIRCULATIONAHA.112.122556 23129601

[18] Chi H Messas E Levine RA Graves DT Amar S. Interleukin-1 receptor signaling mediates atherosclerosis associated with bacterial exposure and/or a high-fat diet in a murine apolipoprotein E heterozygote model: pharmacotherapeutic implications. Circulation. (2004) 110:1678–85. 10.1161/01.CIR.0000142085.39015.31 15353494

[19] Chamberlain J Francis S Brookes Z Shaw G Graham D Alp NJ Interleukin-1 regulates multiple atherogenic mechanisms in response to fat feeding. PLoS One. (2009) 4:e5073. 10.1371/journal.pone.0005073 19347044 PMC2661361

[20] Gomez D Baylis RA Durgin BG Newman AAC Alencar GF Mahan S Interleukin-1beta has atheroprotective effects in advanced atherosclerotic lesions of mice. Nat Med. (2018) 24:1418–29. 10.1038/s41591-018-0124-5 30038218 PMC6130822

[21] Alexander MR Moehle CW Johnson JL Yang Z Lee JK Jackson CL Genetic inactivation of IL-1 signaling enhances atherosclerotic plaque instability and reduces outward vessel remodeling in advanced atherosclerosis in mice. J Clin Invest. (2012) 122:70–9. 10.1172/JCI43713 22201681 PMC3248279

[22] Alfaidi M Wilson H Daigneault M Burnett A Ridger V Chamberlain J Neutrophil elastase promotes interleukin-1beta secretion from human coronary endothelium. J Biol Chem. (2015) 290:24067–78. 10.1074/jbc.M115.659029 26269588 PMC4591798

[23] Alfaidi MA Chamberlain J Rothman A Crossman D Villa-Uriol MC Hadoke P Dietary docosahexaenoic acid reduces oscillatory wall shear stress, atherosclerosis, and hypertension, most likely mediated via an IL-1-mediated mechanism. J Am Heart Assoc. (2018) 7(13):e008757. 10.1161/jaha.118.008757 29960988 PMC6064924

[24] Galea J Armstrong J Gadsdon P Holden H Francis SE Holt CM. Interleukin-1 beta in coronary arteries of patients with ischemic heart disease. Arterioscler Thromb Vasc Biol. (1996) 16:1000–6. 10.1161/01.atv.16.8.1000 8696938

[25] Alfaidi M Acosta CH Wang D Traylor JG Orr AW. Selective role of Nck1 in atherogenic inflammation and plaque formation. J Clin Invest. (2020) 130:4331–47. 10.1172/JCI135552 32427580 PMC8011212

[26] Ghimire K Zaric J Alday-Parejo B Seebach J Bousquenaud M Stalin J MAGI1 Mediates eNOS activation and NO production in endothelial cells in response to fluid shear stress. Cells. (2019) 8(5):388. 10.3390/cells8050388 31035633 PMC6562810

[27] Tsao CW Aday AW Almarzooq ZI Anderson CAM Arora P Avery CL Heart disease and stroke statistics-2023 update: a report from the American heart association. Circulation. (2023) 147(8):e93–621. 10.1161/CIR.0000000000001123 36695182 PMC12135016

[28] Alfaidi M Bhattarai U Orr AW. Nck1, but not Nck2, mediates disturbed flow-induced p21-activated kinase activation and endothelial permeability. J Am Heart Assoc. (2020) 9:e016099. 10.1161/JAHA.120.016099 32468886 PMC7428973

[29] Alfaidi M Wilson H Daigneault M Burnett A Ridger V Chamberlain J Neutrophil elastase promotes interleukin-1β secretion from human coronary endothelium. J Biol Chem. (2015) 290(40):24067–78. jbc.M115.659029 26269588 10.1074/jbc.M115.659029 PMC4591798

[30] Ritchie ME Phipson B Wu D Hu Y Law CW Shi W. Smyth GK. Limma powers differential expression analyses for RNA-sequencing and microarray studies. Nucleic Acids Res. (2015) 43:e47. 10.1093/nar/gkv007 25605792 PMC4402510

[31] Blighe K Rana S Lewis M. Enhancedvolcano: publication-ready volcano plots with enhanced colouring and labeling. R Package Version. (2019) 1. Available at: https://github.com/kevinblighe/EnhancedVolcano

[32] Kolde R. Pheatmap: pretty heatmaps. R Package Version. (2012) 1:726. Available at: https://github.com/raivokolde/pheatmap

[33] Hao Y Hao S Andersen-Nissen E Mauck WM 3rd Zheng S Butler A Integrated analysis of multimodal single-cell data. Cell. (2021) 184:3573–87 e3529. 10.1016/j.cell.2021.04.048 34062119 PMC8238499

[34] Stary HC. Composition and classification of human atherosclerotic lesions. Virchows Arch A Pathol Anat Histopathol. (1992) 421:277–90. 10.1007/BF01660974 1413492

[35] Ayari H Bricca G. Identification of two genes potentially associated in iron-heme homeostasis in human carotid plaque using microarray analysis. J Biosci. (2013) 38:311–5. 10.1007/s12038-013-9310-2 23660665

[36] Chen PY Qin L Baeyens N Li G Afolabi T Budatha M Endothelial-to-mesenchymal transition drives atherosclerosis progression. J Clin Invest. (2015) 125:4514–28. 10.1172/JCI82719 26517696 PMC4665771

[37] Hahn C Schwartz MA. The role of cellular adaptation to mechanical forces in atherosclerosis. Arterioscler Thromb Vasc Biol. (2008) 28:2101–7. 10.1161/ATVBAHA.108.165951 18787190 PMC2737679

[38] Hahn C Schwartz MA. Mechanotransduction in vascular physiology and atherogenesis. Nat Rev Mol Cell Biol. (2009) 10:53–62. 10.1038/nrm2596 19197332 PMC2719300

[39] Chen PY Schwartz MA Simons M. Endothelial-to-Mesenchymal transition, vascular inflammation, and atherosclerosis. Front Cardiovasc Med. (2020) 7:53. 10.3389/fcvm.2020.00053 32478094 PMC7232582

[40] Moonen JR Lee ES Schmidt M Maleszewska M Koerts JA Brouwer LA Endothelial-to-mesenchymal transition contributes to fibro-proliferative vascular disease and is modulated by fluid shear stress. Cardiovasc Res. (2015) 108:377–86. 10.1093/cvr/cvv175 26084310

[41] Mahmoud MM Serbanovic-Canic J Feng S Souilhol C Xing R Hsiao S Shear stress induces endothelial-to-mesenchymal transition via the transcription factor snail. Sci Rep. (2017) 7:3375. 10.1038/s41598-017-03532-z 28611395 PMC5469771

[42] Welch-Reardon KM Wu N Hughes CC. A role for partial endothelial-mesenchymal transitions in angiogenesis? Arterioscler Thromb Vasc Biol. (2015) 35:303–8. 10.1161/ATVBAHA.114.303220 25425619 PMC4911209

[43] Kovacic JC Mercader N Torres M Boehm M Fuster V. Epithelial-to-mesenchymal and endothelial-to-mesenchymal transition: from cardiovascular development to disease. Circulation. (2012) 125:1795–808. 10.1161/CIRCULATIONAHA.111.040352 22492947 PMC3333843

[44] Souilhol C Harmsen MC Evans PC Krenning G. Endothelial-mesenchymal transition in atherosclerosis. Cardiovasc Res. (2018) 114:565–77. 10.1093/cvr/cvx253 29309526

[45] Lecce L Xu Y V'Gangula B Chandel N Pothula V Caudrillier A Histone deacetylase 9 promotes endothelial-mesenchymal transition and an unfavorable atherosclerotic plaque phenotype. J Clin Invest. (2021) 131(15):e131178. 10.1172/JCI131178 34338228 PMC8321575

[46] Lamouille S Xu J Derynck R. Molecular mechanisms of epithelial-mesenchymal transition. Nat Rev Mol Cell Biol. (2014) 15:178–96. 10.1038/nrm3758 24556840 PMC4240281

[47] Dinarello CA. Immunological and inflammatory functions of the interleukin-1 family. Annu Rev Immunol. (2009) 27:519–50. 10.1146/annurev.immunol.021908.132612 19302047

[48] Vromman A Ruvkun V Shvartz E Wojtkiewicz G Santos Masson G Tesmenitsky Y Stage-dependent differential effects of interleukin-1 isoforms on experimental atherosclerosis. Eur Heart J. (2019) 40:2482–91. 10.1093/eurheartj/ehz008 30698710 PMC6685323

[49] Kirii H Niwa T Yamada Y Wada H Saito K Iwakura Y Lack of interleukin-1beta decreases the severity of atherosclerosis in ApoE-deficient mice. Arterioscler Thromb Vasc Biol. (2003) 23:656–60. 10.1161/01.ATV.0000064374.15232.C3 12615675

[50] Bhaskar V Yin J Mirza AM Phan D Vanegas S Issafras H Monoclonal antibodies targeting IL-1 beta reduce biomarkers of atherosclerosis in vitro and inhibit atherosclerotic plaque formation in apolipoprotein E-deficient mice. Atherosclerosis. (2011) 216:313–20. 10.1016/j.atherosclerosis.2011.02.026 21411094

[51] Ridker PM. Anti-inflammatory therapy for atherosclerosis: interpreting divergent results from the CANTOS and CIRT clinical trials. J Intern Med. (2019) 285:503–9. 10.1111/joim.12862 30472762

[52] Ridker PM. Anticytokine agents: targeting interleukin signaling pathways for the treatment of atherothrombosis. Circ Res. (2019) 124:437–50. 10.1161/CIRCRESAHA.118.313129 30702995 PMC6386195

[53] Hettwer J Hinterdobler J Miritsch B Deutsch MA Li X Mauersberger C Interleukin-1beta suppression dampens inflammatory leucocyte production and uptake in atherosclerosis. Cardiovasc Res. (2022) 118:2778–91. 10.1093/cvr/cvab337 34718444 PMC9586563

[54] Shemesh S Kamari Y Shaish A Olteanu S Kandel-Kfir M Almog T Interleukin-1 receptor type-1 in non-hematopoietic cells is the target for the pro-atherogenic effects of interleukin-1 in apoE-deficient mice. Atherosclerosis. (2012) 222:329–36. 10.1016/j.atherosclerosis.2011.12.010 22236482

[55] Nam D Ni CW Rezvan A Suo J Budzyn K Llanos A A model of disturbed flow-induced atherosclerosis in mouse carotid artery by partial ligation and a simple method of RNA isolation from carotid endothelium. J Vis Exp. (2010) (40):1861. 10.3791/1861 20613706 PMC3153900

[56] Collaborators GBDCoD. Global, regional, and national age-sex-specific mortality for 282 causes of death in 195 countries and territories, 1980-2017: a systematic analysis for the global burden of disease study 2017. Lancet. (2018) 392:1736–88. 10.1016/S0140-6736(18)32203-7 30496103 PMC6227606

[57] Libby P Ridker PM Hansson GK. Leducq transatlantic network on A. Inflammation in atherosclerosis: from pathophysiology to practice. J Am Coll Cardiol. (2009) 54:2129–38. 10.1016/j.jacc.2009.09.009 19942084 PMC2834169

[58] Libby P Ridker PM Hansson GK. Progress and challenges in translating the biology of atherosclerosis. Nature. (2011) 473:317–25. 10.1038/nature10146 21593864

[59] Gupta KK Ali S Sanghera RS. Pharmacological options in atherosclerosis: a review of the existing evidence. Cardiol Ther. (2019) 8:5–20. 10.1007/s40119-018-0123-0 30543029 PMC6525235

[60] Dai G Kaazempur-Mofrad MR Natarajan S Zhang Y Vaughn S Blackman BR Distinct endothelial phenotypes evoked by arterial waveforms derived from atherosclerosis-susceptible and -resistant regions of human vasculature. Proc Natl Acad Sci U S A. (2004) 101:14871–6. 10.1073/pnas.0406073101 15466704 PMC522013

[61] Smith SA Samokhin AO Alfadi M Murphy EC Rhodes D Holcombe WML The IL-1RI co-receptor TILRR (FREM1 isoform 2) controls aberrant inflammatory responses and development of vascular disease. JACC Basic Transl Sci. (2017) 2:398–414. 10.1016/j.jacbts.2017.03.014 28920098 PMC5582195

[62] Libby P. Interleukin-1 beta as a target for atherosclerosis therapy: biological basis of CANTOS and beyond. J Am Coll Cardiol. (2017) 70:2278–89. 10.1016/j.jacc.2017.09.028 29073957 PMC5687846

