# Supplementary material for: The interleukin-1 receptor type-1 in disturbed flow-induced endothelial mesenchymal activation
Source: Front Cardiovasc Med. 2023 Jul 19;10:1190460. doi: 10.3389/fcvm.2023.1190460 (PMC10394702; doi:10.3389/fcvm.2023.1190460)
Supplement: Supplementary file 1 [file Table1.docx]

Supplementary Material

# Supplementary Table I: All primary antibodies used in this paper and their stock and final concentrations / dilutions:

| Primary antibody | Spp. | Reactivity | Company | Catalogue No. | Stock Conc./volumes | Final Conc./dilutions |
| --- | --- | --- | --- | --- | --- | --- |
| Anti-hIL-1RI | Mouse MAB | Human | R&D | MAB269 | 100µg | 1/1000 |
| Anti-IL1R1 clone 40101 | Mouse MAB | Human | Abnova | MAB12554 | 50µg | 1/500 |
| Anti-Ncadherin | Rabbit PAB | Human / Mouse | abcam | ab18203 | 1mg/ml | 1/1000 |
| Anti-Actin SMA-FITC antibody CLONE 1a4 | Mouse MAB | Mouse/ Human | Sigma | F3777 | 0.2ml | 1/1000 |
| Anti-CD31 | Mouse MAB | Human / Mouse | abcam | ab9498 | 1mg/ml | 1/1000 |
| Anti-snail1 | Mouse MAB | Human/Mouse | CST | 3895S | 100µl | 1/1000 |
| Anti-Human VE-cadherin (clone # 123413) | Mouse MAB | Human | R&D | MAB9381 (Lot. KCL0322081) | 0.5mg/ml | 1/1000 |
| Anti-Mouse VE-Cadherin | Goat PAB | Mouse | R&D | AF1002-SP | 0.2mg/ml | 1/1000 |
| Anti-GAPDH Antibody | Rabbit MAB | Human/ Mouse | CST | 4436S | 100µl | 1/1000 |

*Abbreviations*: MAB; monoclonal antibody, PAB; polyclonal antibody, IL-1R1; interleukin-1 receptor type I, SMA; smooth muscle actin.
