# Supplementary figures and images for: The interleukin-1 receptor type-1 in disturbed flow-induced endothelial mesenchymal activation
Source: Front Cardiovasc Med. 2023 Jul 19;10:1190460. doi: 10.3389/fcvm.2023.1190460 (PMC10394702; doi:10.3389/fcvm.2023.1190460)

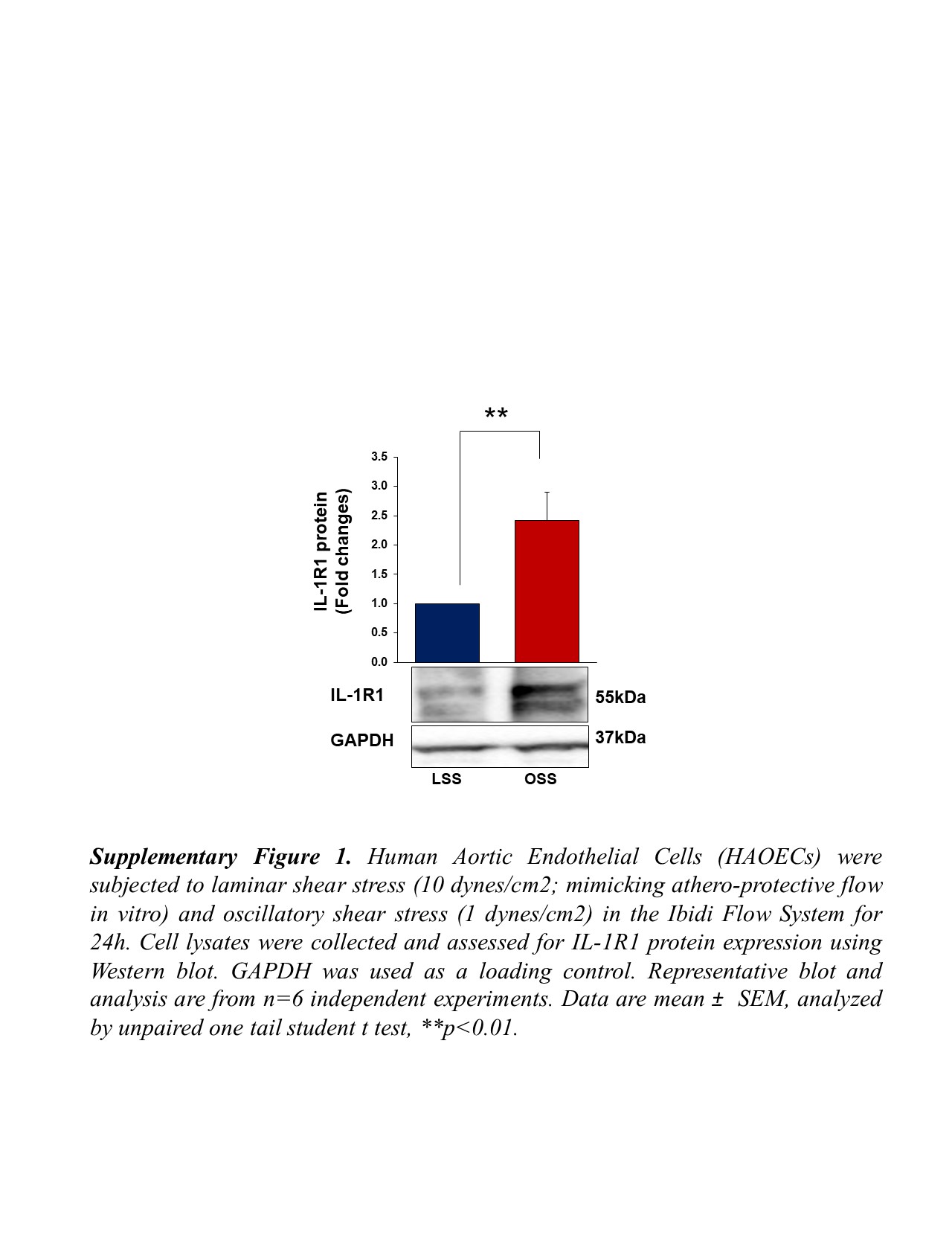

Supplement: Supplementary file 2 [file Image1.jpeg]

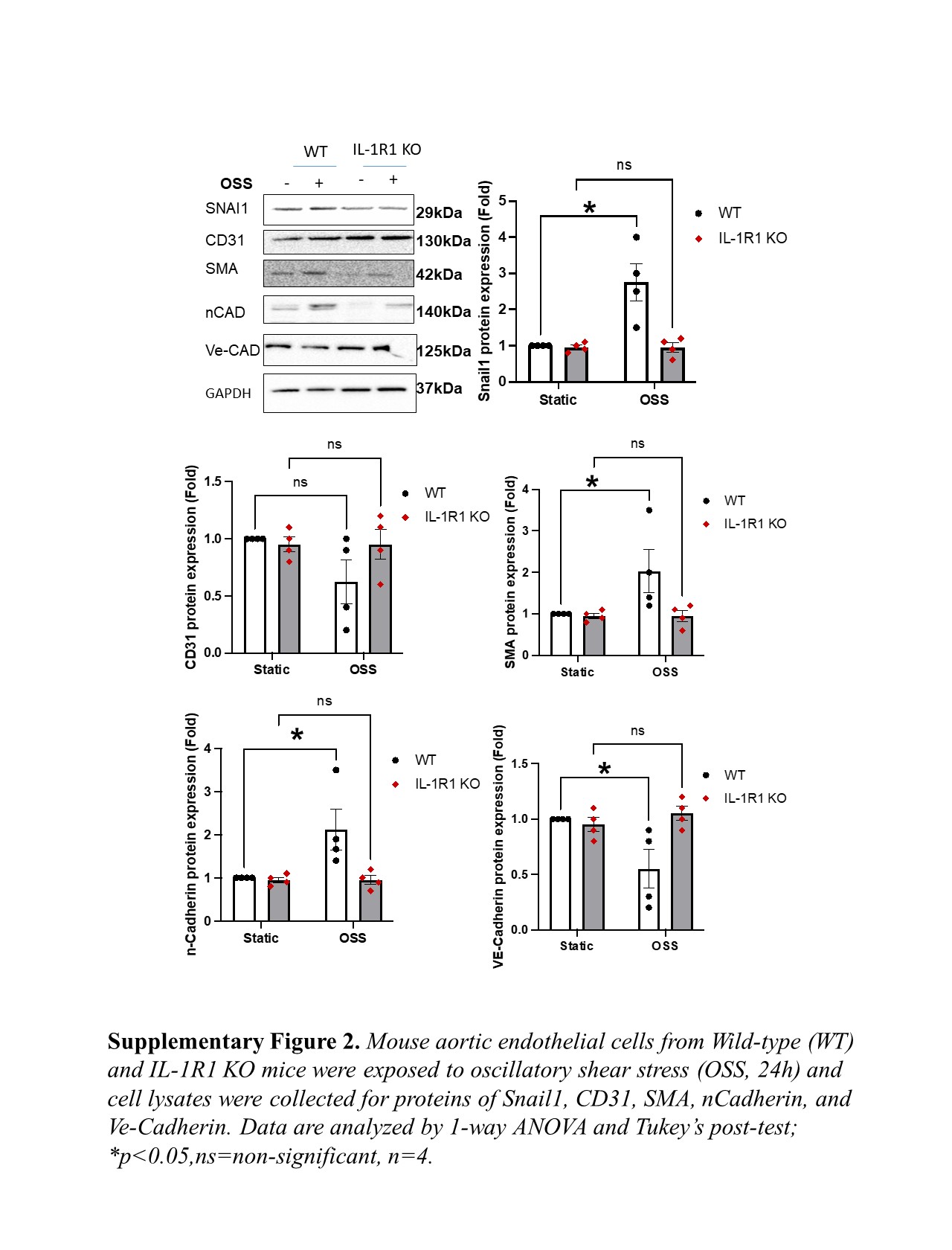

Supplement: Supplementary file 3 [file Image2.jpeg]

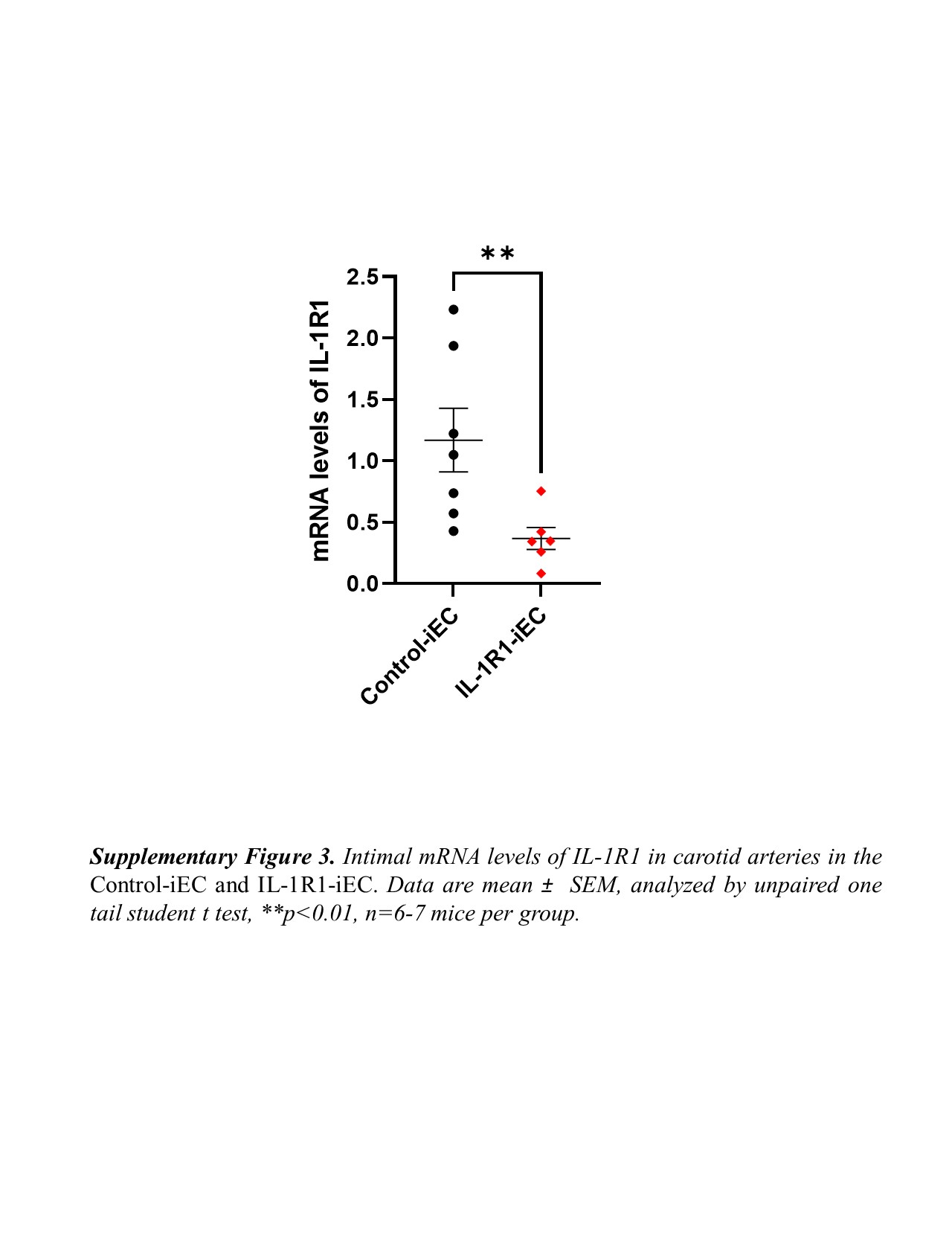

Supplement: Supplementary file 4 [file Image3.jpeg]
